# Origins Matter: Culture Impacts Cognitive Testing in Parkinson’s Disease

**DOI:** 10.3389/fnhum.2019.00269

**Published:** 2019-08-08

**Authors:** Marta Statucka, Melanie Cohn

**Affiliations:** ^1^Krembil Brain Institute, Toronto Western Hospital UHN, Toronto, ON, Canada; ^2^Department of Psychology, University of Toronto, Toronto, ON, Canada

**Keywords:** neuropsychology, visuospatial, executive function, memory, cultural bias, human development index, mild cognitive impairment

## Abstract

Cognitive decline is common in Parkinson’s disease (PD), and precise cognitive assessment is important for diagnosis, prognosis, and treatment. To date, there are no studies in PD investigating cultural bias on neuropsychological tests. Clinical practice in multicultural societies such as, Toronto Canada where nearly half of the population is comprised of first generation immigrants, presents important challenges as most neuropsychological tools were developed in Anglosphere cultures (e.g., USA, UK) and normed in more homogeneous groups. We examine total scores and rates of deficits on tests of visuoperceptual/visuospatial, attention, memory, and executive functions in Canadians with PD born in Anglosphere countries (*n* = 248) vs. in Canadians with PD born in other regions (International group; *n* = 167). The International group shows lower scores and greater rates of deficits on all visuoperceptual and some executive function tasks, but not on attention or memory measures. These biases are not explained by demographic and clinical variables as groups were comparable. Age at immigration, years in Canada, and English proficiency also do not account for the observed biases. In contrast, group differences are strongly mediated by the Historical Index of Human Development of the participants’ country of birth, which reflects economic, health, and educational potential of a country at the time of birth. In sum, our findings demonstrate lasting biases on neuropsychological tests despite significant exposure to, and participation in, Canadian culture. These biases are most striking on visuoperceptual measures and non-verbal executive tasks which many clinicians still considered to be “culture-fair” despite the growing evidence from the field of cross-cultural neuropsychology to the contrary. Our findings also illustrate that socio-development context captures important aspects of culture that relate to cognition, and have important implications for clinical practice.

## Introduction

While basic cognitive processes are often considered universal (Nell, 1999), clinical neuropsychologists and cognitive neuroscientists recognize that a person’s culture impacts how these processes are expressed in behavior such as in their performance on neuropsychological tests (for review, see Puente and Agranovich, 2003; Rivera Mindt et al., 2010; Fernández and Abe, 2018). Because culture also influences the design of cognitive tests (Cole, 1998), it is not surprising that people born and raised where tests are conceived have an advantage. Indeed, what constitutes an average score for well-known cognitive tests (for example the Wechsler scales) varies considerably across different regions of the world. Most tests are developed, standardized, and normed in the United States of America (USA) and United Kingdom (UK), and these two countries are not only predominantly English-speaking, but also share cultural and historical roots and similar high levels of economic and social development. These similarities also extend to other “Anglosphere” countries (a term we borrow from the writer Neal Stephenson), such as Canada, Australia and New Zealand. Cultural biases on cognitive testing are not only evident between disparate geographical regions, but also arise within multicultural societies. However, most of this research has been conducted in the USA and focused on differences between racial and ethnic groups, which is confounded by other group differences such as educational attainment, literacy, English proficiency, and socioeconomic status (Chin et al., 2012; Cagigas and Manly, 2014; Krch et al., 2015; Flores et al., 2017; Weuve et al., 2018). While such research is important, it may not generalize to first-generation immigrants living in multicultural societies as new immigrants face several unique issues which do not necessarily reflect the above confounds (for review, see Ferraro, 2016).

To address cultural biases on cognitive testing, strategies have included collecting normative data for specific groups or countries and adapting existing tests. While a worthwhile endeavor, these strategies do not resolve the challenges of assessing cognition in immigrants at different stages of acculturation. It is not feasible to develop normative data for all subgroups of individuals (Shuttleworth-Edwards, 2016), especially since cultural context is dynamic and transforms from contacts with other cultures and from particular social, historical, and political contexts (Whaley and Davis, 2007). Another strategy is to identify or develop tests that are “culture-fair,” but many efforts have focused on merely avoiding verbal tasks which have proven unsuccessful in eliminating cultural bias (Marcinkowska and Sitek, 2017; Fernández and Abe, 2018). We argue that prior to developing new instruments for use in multicultural settings, we must investigate the degree of bias on existing tasks as it may vary across instruments and cognitive domains, and identify associated features and sources of this bias.

In many ways, Toronto Canada is an ideal location to investigate multicultural bias given that 49% of Torontonians are first-generation immigrants born outside Canada, and 45% identify some language other than English as their mother tongue (Statistics Canada, 2017). Moreover, 50% of Toronto’s immigrants entered the country under the “economic” status meaning that they are generally well-educated and were granted entry into Canada due to their ability to contribute to the Canadian economy [e.g., occupation meets labor market needs, ability to own a business, ability to make substantial investments (Statistics Canada, 2017)]. Immigrants to Canada are also healthier than Canadian-born individuals based on rates of mortality (Ng, 2011) and of chronic conditions such as diabetes and cardiovascular conditions (Newbold and Filice, 2006). These last facts are important because they address some of the criticisms of cross-cultural neuropsychological research in the USA where race/culture is highly confounded with socioeconomic status, educational inequality and health (Rosselli and Ardila, 2003; Schwartz et al., 2004; Chin et al., 2012; Krch et al., 2015; Ferraro, 2016; Weuve et al., 2018).

In the present study, we examine cultural bias in advanced Parkinson’s disease (PD). While this patient group was selected for convenience given the availability of a rich neuropsychological dataset at our center, such investigation is particularly relevant in this patient group where cognitive decline is very common (Emre et al., 2007; Litvan et al., 2012), and the presence of severe cognitive impairment or dementia may preclude access to advanced therapies such as deep brain stimulation (DBS; Lang et al., 2006). As such, if testing is biased, it has the potential to result in health and treatment access inequities. To our knowledge, the effect of cultural diversity has not yet been investigated in this clinical group. In a large cohort of PD patients, we examine whether the frequency of cognitive diagnoses (PD mild cognitive impairment and dementia) differ between people born in Anglosphere countries (Canada, USA, UK), where tests are predominantly developed and normed, relative to individuals born outside these countries (International group), based on clinical interviews and comprehensive neuropsychological testing. On a subset of 12 neuropsychological tests from these assessments, we examine whether the Anglosphere group has higher performance/lower rates of deficits relative to the International group. These tests sample four cognitive domains, namely attention, memory, visuoperceptual/visuospatial skills, and executive functioning. To identify potential sources of bias, we first compare groups’ demographic (i.e., age, sex), socio-economic status (i.e., education, occupational attainment) and clinical characteristics (i.e., disease severity). These demographic and disease-related variables are examined to ensure that the groups are comparable on variables known to impact cognition (e.g., older age, severe PD, and lower education are associated with poorer cognition) so that any between-group differences can be more confidently attributed to sociocultural factors. On biased tasks only, we then investigate whether performance in the International group is associated with immigration variables (e.g., years in Canada, age at immigration) and coarse measures of English proficiency (e.g., English as a mother-tongue, use of interpreter). Last, we investigate whether the relationship between group membership (Anglosphere vs. International) and cognitive performance is mediated by socio-development levels of countries of origin as measured using the Historical Index of Human Development (HIHD; Prados de la Escosura, 2015). The HIHD is an extension of the United Nations Human Development Index (UN-HDI; United Nations Development Programme, 1990) that includes data corresponding to our participants’ country and year of birth. It evaluates countries’ development and well-being beyond economic growth alone, in a scalable and multidimensional manner. Although it does not reflect all aspects of culture, it captures societal factors that facilitate an individual’s growth as it represents people’s ability to access resources (i.e., longevity, education, standard of living).

## Materials and Methods

### Participants

With approval from the research ethics board of the University Health Network (UHN), we conducted a retrospective chart review of advanced PD patients evaluated to determine their candidacy for DBS surgery at Toronto Western Hospital UHN between September 2014 and December 2018. Their multidisciplinary evaluation included a comprehensive neuropsychological assessment and a neurological assessment. Clinical neuropsychologists (M.C. or M.S.) supervised all psychometric testing, conducted clinical interviews, and assigned cognitive diagnoses. The motor examinations were completed by Movement Disorders neurologists. After excluding 40 of the 455 consecutive patients assessed due to other neurological conditions (e.g., prior stroke, TBI with loss of consciousness, epilepsy, prior neurosurgical intervention) or due to incomplete neuropsychological assessments (i.e., missing more than three of the neuropsychological tests of interest), a total of 415 patients were included. Of these, 248 are individuals born in Canada, the USA, and the UK (Anglosphere group), and 167 were born outside these countries (International group). Most participants in the latter group were born in Asia (55%), followed by Europe (23%), the Americas/Caribbean (14%), and Africa (7%), and none were born in Oceania. These proportions are consistent with the general immigrant population of the Toronto Census Metropolitan Area (Statistics Canada, 2017). The number of participants per specific country and world region is presented in Supplementary Table S1.

### Socio-demographic and Disease-Related Variables

Socio-demographic variables include current age, sex, years of formal education, and highest occupation category based on the International Standard Classification of Occupations (International Labour Office, 2012), which includes: (1) managers; (2) professionals; (3) technicians, associate professionals, and clerical workers; (4) craft and trades; (5) services and sales workers; and (6) operators, assemblers, and elementary occupations. Some of the ISCO-08 categories are combined for office workers (technicians and associate professionals class combined with clerical workers) and factory workers (operators and assemblers class combined with elementary occupations), as some positions can be assigned to different categories based on the occupation responsibilities and level of specialization required and this level of detail was not available. If multiple occupations were reported for an individual, the more specialized occupation was coded irrespective of the country in which it was performed. Variables of disease severity include disease duration (years), levodopa equivalence daily dose (LEDD; Tomlinson et al., 2010), motor scores on the Unified Parkinson’s Disease Rating Scale part 3 (UPDRS part 3; Fahn and Elton, 1987). ON and OFF medications, and % levodopa response [(UPDRS part 3 ON—UPDRS part 3 OFF)/UPDRS part 3 OFF]. Some participants (*n* = 57) were evaluated using the new Movement Disorders Society (MDS)-UPDRS part 3 (Goetz et al., 2008), and their scores were transformed to be equivalent to the older version (−7 pts or score of 0 if negative; Hentz et al., 2015). Of note, greater disease severity is reflected by high scores on the UPDRS part 3, high LEDD, and longer disease duration. Additional disease-related variables relate to cognitive diagnoses in PD. Specifically, M.C. and M.S. applied the MDS diagnostic criteria for PD Mild Cognitive Impairment (PD-MCI; Litvan et al., 2012) and PD Dementia (PDD; Emre et al., 2007) based on participants’ full neuropsychological assessment. PD-MCI diagnosis requires self- or family-report of progressive cognitive decline but preserved independence with daily life function, and poor performance (i.e., 1.5 SD below normative mean) on at least two neuropsychological tests. In contrast, PDD requires impairments in at least two domains of cognition, and loss of daily functioning due to cognitive decline.

### Neuropsychological Measures

Although the neuropsychological test battery varied across patients, we selected a subset of measures administered that were common to most assessments and sampled visuoperceptual/visuospatial skills, attention, memory, and executive functioning. Core language skills such as naming and vocabulary are not included as they are not consistently assessed in the International group due to variable language proficiency and well-known cultural bias. Although we recognize that cognitive tasks can tap multiple cognitive domains, we list them here according to their typical classification in clinical neuropsychology. Visuoperceptual/visuospatial measures include the item-response theory version of the Benton Judgement of Line Orientation (JLO; Benton et al., 1983; Spencer et al., 2013), Object Decision and Silhouettes subtests of the Visual Object and Space Perception battery (VOSP; Warrington and James, 1991), and the copy of Rey-Osterrieth Complex Figure (ROCF; Meyers and Meyers, 1995). *Attention* was assessed using the Digit Span subtest of the Wechsler Adult Intelligence Scale 3rd edition (WAIS-III; Wechsler, 1997). *Memory* measures include Total Recall (immediate recall of trials 1–5) and Long Delay Free Recall (LDFR) on the California Verbal Learning Test 2nd edition (CVLT-II; Delis et al., 2000), as well as Recognition on the ROCF (Meyers and Meyers, 1995). We selected the ROCF Recognition over the ROCF free recall trials for our analyses as it has no motor or visuoconstruction component, and as such, provides a purer memory measure. *Executive functioning* measures include errors on the Conditional Associative Learning Test (CALT; Taylor et al., 1990), Matrix Reasoning subtest of the Wechsler Abbreviated Scale of Intelligence 2nd edition (WASI-II; Wechsler, 2011), Category Fluency (Animals and Boys Names) from the Delis Kaplan Executive Function System (DKEFS; Delis et al., 2001), and errors on the Wisconsin Card Sorting Test (WCST; Heaton et al., 1993). Administration of the WCST was discontinued for 55 individuals who achieved zero categories at the midpoint (64 cards) as per administration rules, and their number of errors was doubled to be comparable to scores of individuals who completed the full test. Of these tasks, four measures involve verbal material (Digit Span, Category Fluency, and CVLT-II Total Recall and LDFR). For each measure, we derived two key variables: (1) the total raw scores; and (2) the frequency of impairment defined as scores falling at or below 1.5SD or 6th cumulative percentile relative to age-education corrected normative data for the ROCF and WCST errors [full version (Heaton et al., 1993) or WCST 64 norms for 55 individuals (Kongs et al., 2000)], and relative to age-corrected normative data for the remaining tests.

### Societal and Immigration Variables

For the International group, immigration variables include age at immigration and years in Canada. As coarse measures of English proficiency, mother-tongue (includes English or not), and whether the neuropsychological assessment was completed with the assistance of a professional interpreter were also coded. The option of completing the assessment with an interpreter is offered to all patients who did not complete any part of their schooling in English. Typically, interpretation services were provided in situations wherein individuals have limited or no English proficiency, or when requested by patients. The degree of assistance varies; interpreters may provide clarifications only, adapt and administer tasks with verbal materials in the individual’s preferred language (e.g., Digit Span, CVLT-II, Category Fluency), or provide complete translation of all test instructions and test materials.

For participants in both the Anglosphere and International group, a socio-development context variable is assigned, namely the Historical Index of Human Development (HIHD; Prados de la Escosura, 2015). The HIHD is a historical extension of the United Nation Human Development Index (UN-HDI;United Nations Development Programme, 1990) which is a summary measure of average achievement in key dimensions of human development including health (life expectancy), education (literacy and school enrollment) and standard of living (gross domestic product *per capita* at purchasing power parity). The HIHD value is a number between 0 and 1 with the highest scores representing higher achievement on these combined dimensions. An HIHD score is obtained for each individual based on their country of birth at their year of birth. HIHD values are not available during World War II (WWII). Therefore, any individual born between 1938 and 1944 was assigned their country’s 1938 HIHD value (*n* = 33; 27 Anglosphere, 6 International). Any individual born between January 1945 and June 1952 (post-WWII) was assigned their country’s 1950 HIHD value (*n* = 144; 93 Anglosphere, 51 International). From 1950 onwards, HIHD data is available in 5-year intervals (i.e., 1950, 1955, 1960, etc.). Individuals born 2.5 years before or 2.5 years after a given year were assigned their country’s value for that year (e.g., born between July 1957 and June 1962, assigned value from 1960).

## Statistical Analyses

All analyses were performed using SPSS v. 22. Differences between the Anglosphere and the International groups were assessed with the non-parametric, Mann-Whitney *U* test based on ranks for demographic variables, disease-related variables, neuropsychological test raw scores, and societal variables. This non-parametric test was selected because some variables were not normally distributed. We used chi-square to compare groups’ frequency of impaired neuropsychological test scores. Analyses of the performance on the 12 neuropsychological measures were corrected for multiple comparisons using Bonferroni (*p* < 0.004 or *p* < 0.05 corrected for 12 comparisons). Significance level was uncorrected (*p* < 0.05) for analyses of group demographic and disease-related variables.

For measures showing a significant difference between the Anglosphere and the International groups on both the total score and the frequency of impairment, we carried out further analyses to identify related features and sources of this bias. First, we examined whether age at immigration and years in Canada are related to total score performance in the International group only using Spearman correlations corrected for multiple comparisons (*p* < 0.002 or *p* < 0.05 corrected for 12 comparisons). Second, we investigated the contribution of English proficiency by comparing the total score performance between participants in the International group based on whether English is a mother-tongue, and whether they were tested with or without an interpreter using Mann-Whitney *U* corrected for multiple comparisons (*p* < 0.008 or *p* < 0.05 corrected for six comparisons). For the latter analyses, in cases where significant difference are noted, we also verified whether these remained after controlling for demographic variables that differed between subgroups.

Third, we investigated whether the relationships between group (Anglosphere vs. International) and performance are mediated by socio-development context (HIHD). Mediations analyses were selected because HIHD and group membership are collinear (VIF > 5), and hence, not appropriate for multiple regression models. We used PROCESS v.3.3^1^ (Hayes, 2017) implemented in SPSS to test our mediation models. We used bootstrapping (5,000 resampling) with 95% confidence intervals to test whether the mediated models are significantly different from the direct models. Any confidence interval that did not include 0 was considered significant. Because the WCST errors showed a bimodal distribution of residuals, this variable was not analyzed further using mediation models.

## Results

### Demographic and Clinical Characteristics

Socio-demographic and clinical characteristics, presented in Table 1 with related statistics, show that the International and Anglosphere groups are comparable. Indeed, there are no significant group differences in terms of sex and years of education which is high in both groups (*Md* = 14 for both groups). Occupation classification was also not different between groups and demonstrates high occupational achievement in both groups with more than half having been employed as managers or professionals. Four of five disease-related measures are not significantly different between groups including the UPDRS part 3 ON, % levodopa response, LEDD, and PD duration. There are also no significant group differences in the frequency of cognitive diagnosis (combined PD-MCI and PDD vs. intact cognition) or frequency of reported cognitive complaint. However, the International group meets the psychometric criteria of the clinical cognitive diagnosis (i.e., 2 or more tests falling 1.5 SD below normative data) more frequently (16.4% difference). This confirmed that psychometric deficits are more commonly observed in the International group than in the Anglosphere group although it does not provide information on the types of deficits.

**Table 1:** Demographic, disease-related, and societal characteristics of Canadian Parkinson’s disease (PD) patients based on their region of birth [Frequency and Median (IQR)].

	Anglosphere (*n* = 248)	International (*n* = 167)	Statistics (*U* for ranks, *χ*^2^ for frequency)
**Socio-demographic**			
Age	63.55 (10.44)	61.77 (11.17)	*U* = 17,492, *p* = 0.007*, *d* = 0.27
Sex (%Female)	32.3%	39.5%	*χ*^2^ = 2.31, *p* = 0.13, *d* = 0.15
Education (years)	14 (4)	14 (4)	*U* = 18,943.5, *p* = 0.14, *d* = 0.15
Highest Occupation (ISCO-08)^b^			$\chi_{\left(5\right)}^{2}$ = 2.33, *p* = 0.80, *v* = 0.08
- Managers	29.0%	25.7%	
- Professionals	29.8%	29.3%	
- Technicians, associates, clerical	21.4%	19.2%	
- Craft and trades	6.5%	7.8%	
- Services and sales	6.9%	9.0%	
- Operators, assemblers, elementary occupations	6.5%	9.0%	
**Disease-related**			
PD duration	9.22 (5.95)	9.87 (5.48)	*U* = 19,117.5, *p* = 0.18, *d* = 0.13
UPDRS part 3 OFF^a^	34 (14.5)	39 (17)	*U* = 16,236.5, *p* = 0.001*, *d* = 0.34
UPDRS part 3 ON	14 (12)	15 (12)	*U* = 18,983.5, *p* = 0.15, *d* = 0.14
% Levodopa response^a^	60 (24)	59 (24)	*U* = 20,180.5, *p* = 0.98, *d* = 0.00
Levodopa equivalent daily dose (LEDD)	1,327 (783)	1,373 (700)	*U* = 19,890, *p* = 0.50, *d* = 0.07
Cognitive complaint	86.7%	80.8%	*χ*^2^ = 2.59, *p* = 0.11, *d* = 0.16
Psychometric	73.4%	89.8%	*χ*^2^ = 16.84, *p* < 0.001*, *d* = 0.41
			(intact vs. MCI + PDD)
Cognitive diagnosis: Intact:MCI:PDD	39.5%:60.1%:0.4%	31.7%:67.1%:1.2%	*χ*^2^ = 2.61, *p* = 0.11, *d* = 0.16
**Societal**			
HIHD	0.49 (0.06)	0.23 (0.18)	*U* = 658, *p* < 0.001*, *d* = 2.88
Age at immigration	–	30.7 (25)	–
Years in Canada	–	27.0 (19)	–
English mother-tongue	94.8%	13.8%	*χ*^2^ = 278.30, *p* < 0.001*, *d* = 2.85
Interpreter assistance	0.4%	32.4%	–

Note: ^a^Sample size Anglosphere = 245 and International = 165 as some individuals did not complete the UPDRS part 3 OFF medications. ^b^ISCO-08, International Standard Classification of Occupations; UPDRS, Unified Parkinson’s Disease Rating Scale; MCI, Mild Cognitive Impairment; PDD, Parkinson’s Disease Dementia; HIHD, Historical Index of Human Development. *Significant results with *p* < 0.05 uncorrected.

A few additional differences appear between groups. The Anglosphere group is older (1.78 years difference) and has a lower UPDRS part 3 OFF score (5 points) relative to the International group. Despite this, these variables are not used as covariates in analyses of cognitive data for simplicity and for the following specific reasons. Age is accounted for in normative scores (i.e., frequency of impairments), its potential effect on raw scores favors the International group and as such does not inflate a possible Type 1 error, and age (year of birth) is used to derive the HIHD. As for the UPDRS part 3 OFF score, this is the only disease-severity variable showing a significant difference and given that participants are tested in the ON state, it is unlikely to affect performance directly.

### Neuropsychological Variables

**Figure 1:**
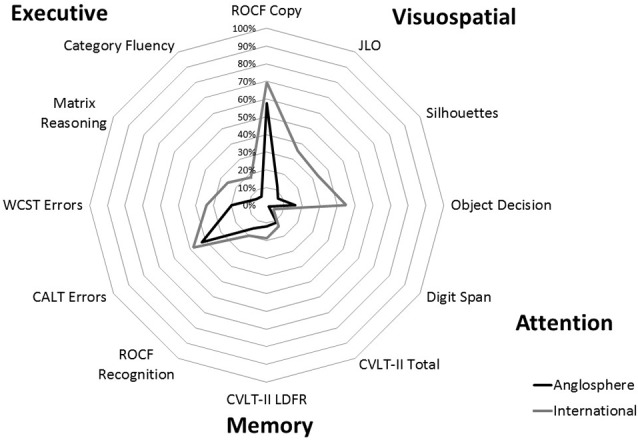
Frequency of impairment per test across Anglosphere and International Parkinson’s disease (PD) groups.

As shown in Table 2 and Figure 1, analyses yield similar finding when comparing total scores (ranks) and frequency of clinically-relevant deficits across groups. Group differences of medium to large effect sizes are observed on six measures (Cohen’s *d* ranging from 0.30 to 0.82, see Table 2 for statistical tests). The International group performs more poorly than the Anglosphere group on all tasks of visuoperception, including JLO, Silhouettes and Object Decision. The International group’s performance is also poorer on measures of executive functioning including Matrix Reasoning, Category Fluency and WCST errors. In contrast, there is no significant group difference (Cohen’s *d* ranging from 0.02 to 0.26) in working memory errors on the CALT, and on measures of attention and memory including Digit Span, CVLT-II Total, CVLT-II LDFR, and ROCF Recognition. There is also no significant difference in complex visuoconstruction on the ROCF copy, although a trend is observed but does not survive correction for multiple comparisons (Cohen’s *d* of 0.20 and 0.24 for ranks and frequency of impairments, respectively).

**Table 2:** Total score and frequency of impairment on neuropsychological tests in Canadian PD patients according to their region of birth.

		Median (IQR)		Deficit frequency		
Cognitive test	Sample size (Angl.:Intern.)	Angl.	Intern.	Angl.	Intern.	Statistics (*U* for ranks, *χ*^2^ for frequency)
*Attention*						
Digit Span	247:166	16 (4)	15 (7)	1.2%	4.2%	*U* = 17,397, *p* = 0.009, *d* = 0.26 *χ*^2^ = 3.79, *p* = 0.052, *d* = 0.19
*Memory*						
CVLT-II Total	248:166	42 (14)	40.5 (14)	11.3%	13.9%	*U* = 18,947.5, *p* = 0.17, *d* = 0.14 *χ*^2^ = 0.61, *p* = 0.44, *d* = 0.08
CVLT-II LDFR	248:166	9 (5)	9 (6)	12.1%	18.7%	*U* = 20,370, *p* = 0.86, *d* = 0.02 *χ*^2^ = 3.43, *p* = 0.06, *d* = 0.18
ROCF recognition	245:161	20 (3)	19 (3)	15.1%	19.9%	*U* = 18,114.5, *p* = 0.16, *d* = 0.14 *χ*^2^ = 1.66, *p* = 0.21, *d* = 0.13
*Visuoperceptual/visuospatial skills*				
JLO	248:167	25 (7)	21 (8)	12.1%	35.3%	*U* = 13,180, *p* < 0.001*, *d* = 0.65 *χ*^2^ = 31.97, *p* < 0.001*, *d* = 0.58
Silhouettes	247:135	21 (5)	18 (7)	7.3%	33.3%	*U* = 9,879, *p* < 0.001*, *d* = 0.72 *χ*^2^ = 42.99, *p* < 0.001*, *d* = 0.71
Object Decision	248:167	17 (3)	15 (4)	16.1%	44.9%	*U* = 11,487.5, *p* < 0.001*, *d* = 0.82 *χ*^2^ = 41.27, *p* < 0.001*, *d* = 0.66
ROCF copy	246:165	29 (8.5)	27 (9.5)	57.7%	69.7%	*U* = 17890, *p* = 0.041, *d* = 0.20 *χ*^2^ = 6.04, *p* = 0.014, *d* = 0.24
*Executive functions*					
Matrix Reasoning	247:164	18 (6)	13 (10)	6.9%	25.6%	*U* = 14,536, *p* < 0.001*, *d* = 0.49 *χ*^2^ = 28.12, *p* < 0.001*, *d* = 0.54
Category fluency	247:166	38 (11)	31 (11)	5.7%	18.1%	*U* = 11,369, *p* < 0.001*, *d* = 0.82 *χ*^2^ = 16.05, *p* < 0.001*, *d* = 0.40
CALT errors	247:165	31 (33)	33 (39.5)	42.1%	47.9%	*U* = 19,353.5, *p* = 0.39, *d* = 0.09 *χ*^2^ = 1.33, *p* = 0.25, *d* = 0.11
WCST errors	247:164	41 (40)	51 (44)	19.8%	34.1%	*U* = 16,725.5, *p* = 0.003*, *d* = 0.30 *χ*^2^ = 10.61, *p* = 0.001*, *d* = 0.33

Note: ^a^CVLT-II, California Verbal Learning Test 2nd edition; LDFR, Long Delay Free Recall; ROCF, Rey-Osterrieth Complex Figure; JLO, Benton Judgement of Line Orientation; CALT, Conditional Associative Learning Test; WCST, Wisconsin Card Sorting Test. *Significant results with *p* < 0.05 corrected for multiple comparisons using Bonferroni (p < 0.004 corrected for 12 comparisons).

### Sources of Cultural Bias

To investigate potential contributing variables to the bias noted on measures of visuoperception and executive functioning, we first examine the relationship between the International group’s total score on each biased measure and immigration variables. As shown in Table 1, the majority of the International group immigrated to Canada in adulthood (*Md* = 30.7, IQR = 25) and has lived in Canada for *Md* = 27 years (IQR = 19). We found no significant correlation (*p* < 0.002–Bonferroni corrected) between performance on the six biased measures and age at immigration (JLO: *r*_s_ = −0.08, *p* = 0.32; Silhouettes: *r*_s_ = −0.14, *p* = 0.10; Object Decision: *r*_s_ = −0.13, *p* = 0.08; Matrix Reasoning: *r*_s_ = 0.03, *p* = 0.74; Category Fluency: *r*_s_ = −0.19, *p* = 0.01; WCST errors: *r*_s_ = 0.08, *p* = 0.29) nor between performance and years in Canada (JLO: *r*_s_ = −0.04, *p* = 0.57; Silhouettes: *r*_s_ = 0.02, *p* = 0.80; Object Decision: *r*_s_ = 0.03, *p* = 0.72; Matrix Reasoning: *r*_s_ = −0.16, *p* = 0.04; Category Fluency: *r*_s_ = 0.04, *p* = 0.64; WCST errors: *r*_s_ = 0.03, *p* = 0.71).

Another potential contributing factor is participants’ proficiency in English. In the Anglosphere group, English is the mother-tongue of 94.8% of participants and only a single participant was tested with an interpreter (0.4%). In contrast, in the International group, English is a mother-tongue in 13.8% of participants, and 32.4% of participants were assessed with the assistance of interpreters. However, within the International group, performance (ranks of total scores) on biased neuropsychological tasks does not differ significantly (*p* < 0.008–Bonferroni correction) between individuals for whom English is a mother-tongue vs. those for whom it is not (JLO: *U* = 1,649, *p* = 0.97, *d* = 0.01; Silhouettes: *U* = 861, *p* = 0.13, *d* = 0.26; Object Decision: *U* = 1,307, *p* = 0.10, *d* = 0.25; Category Fluency: *U* = 1463, *p* = 0.40, *d* = 0.13; Matrix Reasoning: *U* = 1,425.5, *p* = 0.51, *d* = 0.10; WCST errors: *U* = 1280.5, *p* = 0.17, *d* = 0.21). Importantly, these analyses are quite underpowered as the sample of individuals with English as a mother-tongue is small (depending on the test, *n* = 19–23 report English as a mother-tongue vs. *n* = 116–144 report other languages).

The impact of language on performance can also be assessed by comparing the performance (ranks of total scores) of participants from the International group tested with an interpreter (*n* = 55) to those tested without (*n* = 112). Here, we demonstrate no significant difference (*p* < 0.008–Bonferroni correction) in performance on JLO (*U* = 2604, *p* = 0.10, *d* = 0.25), Silhouettes (*U* = 1559.5, *p* = 0.13, *d* = 0.26), Object Decision (*U* = 2486.5, *p* = 0.04, *d* = 0.31) and Category Fluency (*U* = 2672.5, *p* = 0.23, *d* = 0.19), but their performance is weaker on Matrix Reasoning (*U* = 2180.5, *p* = 0.006, *d* = 0.44) and WCST errors (*U* = 2075.5, *p* = 0.001, *d* = 0.52). However, whilst these two subgroups are comparable in terms of age (*U* = 3,003, *p* = 0.79, *d* = 0.04) and disease severity (PD duration: *U* = 3,045, *p* = 0.91, *d* = 0.02; UPDRS part 3 ON: *U* = 2647.5, *p* = 0.14, *d* = 0.23; UPDRS part 3 OFF: *U* = 2,995, *p* = 0.99, *d* = 0.00; % levodopa response: *U* = 2666.5, *p* = 0.30, *d* = 0.18; LEDD: *U* = 2,793, *p* = 0.33, *d* = 0.15), participants tested with interpreters have lower education than participants tested without (*U* = 1980.5, *p* < 0.001, *d* = 0.61). After regressing out years of education, group differences are reduced and below corrected statistical significance (Matrix Reasoning corrected for education: *U* = 2,634, *p* = 0.24, *d* = 0.18; WCST errors corrected for education: *U* = 2,325, *p* = 0.02, *d* = 0.37).

Because the Anglosphere and International groups were comparable on demographic and disease variables, we do not test whether these variables have a differential effect on performance on biased tasks within each group, but these data are presented in Supplementary Tables S2, S3. We also do not analyze differences in performance between the different world regions in the International group, but rates of impairments for each of the 12 neuropsychological measures per global region are presented in Supplementary Figure S1 and show no striking or consistent pattern of regional bias.

### Socio-development Context and Cognition

**Figure 2:**
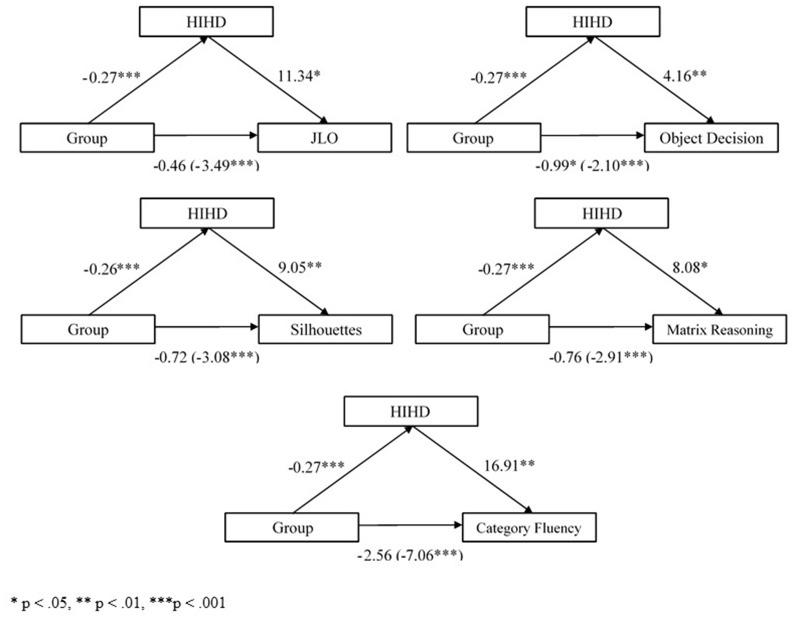
Mediation models for group membership (x), Historical Index of Human Development (HIHD; M), and performance on biased tasks (y).

As predicted, a key difference between the Anglosphere and the International groups pertains to the HIHD which is significantly higher in the Anglosphere group (see Table 1 for statistics). To investigate whether this socio-development context is a source of the bias observed on cognitive measures, we conducted five simple mediation analyses with group membership (x), HIHD (M), and cognitive performance on the biased tasks (Y). As noted previously, WCST errors were not analyzed in these mediation models due to its bimodal distribution of residuals. As shown in Figure 2, HIHD was a full mediator in four of five models and a partial mediator in one. The total effect of group membership on JLO performance was significant [*c* = −3.49, *SE* = 0.54, 95% CI (−4.55, −2.42)]. However, the direct effect of group membership on JLO was completely mediated when HIHD was taken into account [*c*′ = −0.46, *SE* = 1.04, 95% CI (−2.51, 1.59)]. HIHD was also a full mediator in the relationship between group membership and Silhouettes [*c* = −3.08, *SE* = 0.41, 95% CI (−3.89, −2.27); *c*′ = −0.72, *SE* = 0.78, 95% CI (−2.26, 0.82)], group membership and Matrix Reasoning [*c* = −2.91, *SE* = 0.52, 95% CI (−3.95, −1.88); *c*′ = −0.76, *SE* = 1.03, 95% CI (−2.78, 1.26)], and group membership and Category Fluency [*c* = −7.06, *SE* = 0.89, 95% CI (−8.81, −5.31); *c*′ = −2.56, *SE* = 1.72, 95% CI (−5.95, 0.82)]. In the case of Object Decision, the total effect was significant [*c* = −2.10, *SE* = 0.24, 95% CI (−2.57, −1.62)] and this direct effect was significantly lessened or partially mediated by HIHD [*c*′ = −0.99, *SE* = 0.47, 95% CI (−1.91, −0.07), *p* = 0.03].

## Discussion

In advanced PD, we demonstrate a strong cultural bias on psychometric testing favoring individuals born in Anglosphere countries over first-generation immigrants born in other countries. This bias is observed on several measures of visuoperception and executive functioning, including JLO, Silhouettes, Object Decision, WCST, Matrix Reasoning, Category Fluency, and a trend is noted in complex visuoconstruction on the ROCF copy. However, no significant effect of culture is evident on measures of auditory attention (Digit Span), verbal and visual memory (CVLT-II and ROCF recognition), and spatial working memory (CALT). Of the potential contributing factors, disease-related and demographic characteristics do not account for the noted cultural bias as these are similar between groups, and immigration variables and English proficiency also do not relate significantly to performance in the International group. Notably, we demonstrate that the socio-development context specific to the time and place of birth of our participants strongly contributes to cultural bias on cognitive testing. Indeed, the Historical Index of Human Development (HIHD) mediates the relationship between group and performance completely for four biased tasks, and partially for one task.

Together, our findings have important implications for cross-cultural cognitive neuroscience as they demonstrate that culture has differential effects across cognitive domains (with visuoperception being particularly vulnerable), and that the HIHD captures important aspects of this cultural effect. Our results are also highly relevant for clinical neuropsychology practice in PD as well as in other neurological conditions. We discuss these points as well as study limitations in turn.

### Cultural Bias and Cognitive Domains

It may be surprising that some of the most striking, consistent biases are seen on tests of basic visuoperception as well as executive tasks utilizing visual stimuli (WCST, Matrix Reasoning), in which verbal abilities play little or no role, rather than on tasks with a prominent language component (e.g., CVLT-II and Digit Span). This runs counter to common clinical practice in neuropsychology which emphasizes the use of non-verbal tests, presumed to be less biased than verbal tasks, in assessing culturally diverse populations (for review, see Rosselli and Ardila, 2003). In fact, many “culture-fair” tests of general intellectual functioning [e.g., Cattell’s Culture Fair Test (Cattell, 1940), Raven’s Progressive Matrices (Raven and Court, 1998), Test of Nonverbal Intelligence (TONI; Brown et al., 1990), Naglieri Nonverbal Ability Test (NNAT; Naglieri, 2003)] contain non-verbal abstract reasoning tasks similar to the Matrix Reasoning subtest of the Wechsler scales, where we find a significant cultural bias. One factor to be considered when interpreting these results relates to differences in semantic knowledge of the visual stimuli between cultures, an issue which Luria noted following his expedition to Uzbekistan in 1931 (for description, see Nell, 1999). Certainly the two VOSP tasks do utilize common objects whose prototypical form may vary slightly between cultures (e.g., a kettle, a purse). But importantly, our participants have been exposed to Canadian culture and Anglosphere-typical representations of these objects for nearly three decades, and there is no relationship between the number of years in Canada and performance on these tasks. This finding is similar to results showing that years in Denmark did not influence performance on several visuoconstruction tasks (including Clock Drawing) among Turkish immigrants (Nielsen and Jørgensen, 2013). Moreover, it is also difficult to apply this explanation to the JLO where individuals are simply asked to match the angles of two lines, which does not seem to rely on semantic knowledge.

These findings are, however, consistent with a growing cognitive neuroscience literature recognizing that visuospatial abilities are not immune to cultural effects. For example, susceptibility to basic visual illusions, color perception, and visual attention vary between cultures (Masuda, 2009) but it remains unclear to what extent these difference reflect item, method, and/or construct bias of the tasks (Van de Vijver and Tanzer, 2004) vs. underlying neurobiological mechanisms. While our Anglosphere group outperformed the International group on all visuoperceptual tasks, there is evidence that individuals from other cultures do perform better on some experimental tasks tapping different aspects of visuospatial abilities (e.g., mental rotation in Chinese speakers, Li and O’Boyle, 2011; Li et al., 2014). However, we are unaware of any clinical visuospatial tasks where non-Anglosphere individuals have an advantage, and this is likely related to where and how clinical measures are conceived, created, and standardized. Therefore, it seems that experimental findings from the cognitive neurosciences have yet to translate to clinical practice, and this will be key to the development of “culture-fair” tests.

Cultural bias is also observed in Category Fluency, despite the fact that it was administered in the patients’ language of choice and that the open-ended nature of the cues (i.e., Animals and Boys Names) allows the generation of exemplars reflecting participants’ culture. This task has been recommended for use in cross-cultural neuropsychology (Ardila et al., 2006) as comparable performance is observed in older adults across different Spanish-speaking countries (Ostrosky-Solis et al., 2007) and between monolinguals and bilinguals in a multicultural setting (Luo et al., 2010). However, there is also evidence of cultural bias in some immigrant groups, and like our groups, is not related to time since immigration (Nielsen et al., 2012; Nielsen and Waldemar, 2016; Peviani et al., 2016). Perhaps the speeded nature of the task, which is the only timed task analyzed, may be a contributing factor as the emphasis on speed can vary across cultures (Ardila, 2005).

As for other cognitive domains, no significant bias is noted in attention span, working memory, and episodic memory despite the verbal nature of some tasks (Digit Span, CVLT-II), or the visuoperceptual nature of others (ROCF recognition, CALT). The literature is inconsistent with respect to the cultural effect on attention span and working memory, with evidence showing both significant (Ostrosky-Solís and Lozano, 2006) and null effects (Hedden et al., 2002). As for episodic memory, it is generally accepted that core episodic memory abilities are universal given strong evidence that medial temporal lobe (MTL) lesions reliably lead to profound memory loss. Differences in perception and semantic knowledge as noted above can, however, influence the specific characteristics of the stored memory representations (Gutchess and Indeck, 2009), although this did not translate in performance differences on the tasks used here.

### Human Development Index as a Measure of Culture

We found that countries’ Human Development level captures aspects of culture that accounts for a significant proportion of the noted bias on cognitive test performance examined here. This is a novel way to address culture in the field of cross-cultural neuropsychology, and we are aware of only two other studies that have used this variable to explain cognitive test performance. Berg et al. (2017) found that in a sample of young people with psychosis, high values on the original UN-HDI predicted better executive functioning scores among individuals with Norwegian heritage and first-generation immigrants to Norway. These findings are relevant to our current study in a number of ways. First, there is an overlap between the measures used in both studies (e.g., Category Fluency, Matrix Reasoning, and Digit Span) and in findings (e.g., strong relationship between HDI and executive tasks). Second, this study was conducted in Norway, which has a high HDI but is not an Anglosphere country where neuropsychological tests are typically conceived. Moreover, immigrants in this study moved to Norway before school age, completed all of their education within the Norwegian educational system, and were fluent in a Scandinavian language. In contrast, participants in our International group typically immigrated to Canada as adults and thus, completed all or most of their education in their home country. Despite these differences in the potential level of acculturation, age, and disease, both studies demonstrate a relationship between HDI and cognitive test performance suggesting that early life within specific socio-developmental contexts has a strong and long-lasting impact on cognitive testing. The second study demonstrated that Latin Americans’ performance on a widely used test of cognitive effort, the Test of Memory Malingering (TOMM), was strongly correlated with UN-HDI of the eight countries in which they resided (Nijdam-Jones et al., 2017). The authors warn about the cross-cultural applicability of the TOMM and especially the use of North American cut-off scores in other populations. Therefore, though it may seem like a crude measure that ignores intra-country diversity (albeit intra-country HDI metrics do exist for some countries) and is far from encompassing all aspects of a society and its culture, human development nonetheless relates to cognitive test performance and can help elucidate why and how cognitive abilities differ across cultures.

### Relevance to Clinical Neuropsychology

The presence of cultural bias on neuropsychological testing has important implications for the field of neuropsychology at large as psychometric testing is used clinically to diagnose conditions such as MCI and dementia and to determine the degree of neurocognitive compromise across different disorders. For instance, in neurodegenerative disease research, this bias likely impacts epidemiological studies estimating prevalence, and the investigation of biomarkers and risk factors for poor cognitive prognosis. Studies in Alzheimer’s disease (AD) and related MCI illustrate this. Despite controlling for numerous factors, the prevalence of MCI and dementia varies greatly across the world (Sachdev et al., 2015) and across race/ethnic groups within the USA (Katz et al., 2012; Ferraro, 2016). Part of the issue may be related to the use of biased tests and/or inappropriate norms. For example, when North American norms are applied to people from other countries (namely, Morocco, Spain, and Colombia), up to 51% of individuals are misdiagnosed with MCI or dementia, albeit diagnoses in this study were solely based on psychometric data without considering self- or family-reports of cognitive decline (Daugherty et al., 2017). In terms of prognostication, different patterns of cognitive performance may predict eventual conversion to dementia in different ethnic/racial groups (Weissberger et al., 2013). As for biomarkers, a pertinent example is that the correspondence between the degree of MTL atrophy in MCI and the magnitude of cognitive dysfunction on testing varies across race/ethnicity despite controlling for various risk factors (DeCarli et al., 2008; Burke et al., 2018; Weissberger et al., 2019). It is reasonable to assume that cultural bias on testing has a similar impact on MCI and dementia in PD. To our knowledge, there are no studies other than ours addressing this directly, however, a recent study aimed at identifying tests that consistently detect cognitive decline in PD is particularly relevant (Hoogland et al., 2018). Neuropsychological data of 2,908 non-demented PD patients from 20 international studies and nine countries (USA, Canada, New Zealand, Australia, Spain, Italy, Netherland, Germany, and Taiwan) were pooled, and although the cognitive domains affected were consistent with the PD literature (memory, executive dysfunction and attention), no specific tests were recommended due to high between-study variability. It is unclear whether the inclusion of societal context variables (e.g., HIHD) could help reduce this heterogeneity and if so, whether it could be used as a correcting factor in multicultural and international collaborative studies. For example, several authors have used regression models to “correct” test scores for individual differences (i.e., age, education, sex; Cavaco et al., 2013a,b; Casaletto et al., 2016; Abou-Mrad et al., 2017; Alobaidy et al., 2017; Kirsebom et al., 2019). A similar correction approach may be adopted with the HIHD or other societal variables in future studies.

In addition to diagnosis, cultural bias also affects the evidence supporting the identification of risk factors. It is well recognized that research productivity, including that in psychology (Arnett, 2008; Henrich et al., 2010) and neurology (Jamjoom and Jamjoom, 2016), is geographically biased with a strong proportion of published studies coming from the USA and other Anglosphere countries. Within these studies, diversity of research participants is also reduced by a selection bias. This applies to the few studies investigating risk factors for cognitive decline in PD. For instance, a cognitive phenotype consisting of visuospatial deficits and poor category verbal fluency has been associated with rapid progression to PD dementia (Foltynie et al., 2004; Williams-Gray et al., 2007, 2013). This study was completed in Cambridgeshire England, and the patient group was identified to be 98% Caucasian. Given our results showing cultural bias on Category Fluency and visuospatial functioning, the two main characteristics of the “at-risk” phenotype, it is unclear whether performance on these tasks would retain its predictive value in more culturally diverse patient groups.

Importantly, while we demonstrate a significant bias on psychometric tests, it did not translate in increased rates of PD-MCI/dementia because these diagnoses require the presence of subjective cognitive complaints and the frequency of such reported cognitive decline did not differ between our groups. Based on our results, Daugherty et al.’s (2017) findings of elevated misdiagnoses of MCI and dementia in Morocco, Spain and Colombia may be overestimated because reports of cognitive decline were not required for diagnosis assignment. This highlights the importance of not solely relying on psychometric data for diagnostic purpose, but also integrating information on the reported course of cognitive change and their impact on daily function for individual patients. However, it is important to note that individuals’ perception of their own cognition, mood, and general health, and their subjective complaints are not free for cultural bias either (Karasz, 2005; Jürges, 2007; Mograbi et al., 2012; Wu, 2016; Molina, 2017; Rossouw et al., 2018).

### Limitations

In terms of limitations of the current study, several other factors previously identified as contributors to cultural bias on cognitive testing are not accounted for. First, other than education and occupational attainment (in Canada or abroad), other indicators of socio-economic status such as current wealth or income are not available. Although most individuals come to Canada under the economic class category, current poverty remains higher in this group relative to Canadian-born individuals [i.e., chronic low income is 4.8% in Canadian-born vs. 13.2% in Toronto immigrants even 15–20 years after landing (Lu and Picot, 2017)].

Second, because our study is retrospective, we do not have specific measures of English proficiency other than whether English is a first language or whether an interpreter provided assistance with the assessment. It is unclear which measure would be appropriate given linguistic differences across English-speaking countries and regions, and we question whether this would account for the noted group differences given that no bias was found on some tasks involving verbal material (e.g., CVLT-II). Similarly, we do not have information on whether participants in both groups are bi- or multi-lingual and to what degree of proficiency. This may be relevant in light of research, in part conducted in Toronto, on the beneficial effect of bilingualism to executive functioning (Bialystok et al., 2008; Nielsen et al., 2019) and its potential protective effect from age-related cognitive decline (Bialystok et al., 2014).

Third, we also did not measure the degree of acculturation other than by the number of years in Canada, which is frequently used as a proxy of acculturation with the caveat that it does not necessarily reflect cultural incorporation (Fox et al., 2017). Relatedly, we do not consider potential acculturation or cultural effects in the Anglosphere group, which likely includes a high proportion of second-generation immigrants given that this reflects 28% of Toronto’s population (Statistics Canada, 2017). Transgenerational cultural effects have been documented in general health research (Fox et al., 2015) and neuropsychological studies (Kemmotsu et al., 2013; Bossuroy et al., 2014; Ferraro, 2016), thus our study may actually underestimate the cultural effect on cognitive tests. While acculturation is an important factor to consider in future studies, several challenges have been identified in integrating such measures in health research, as illustrated by conflicting findings attributed to inconsistent conceptualization and operationalization across studies (Fox et al., 2017). Measuring acculturation in highly diverse societies such as Toronto is further complicated because it requires consideration of hybrid or fusion culture(s) in addition to the original and host cultures, and as such, the bi-dimensional instruments commonly used are not appropriate (Fox et al., 2017).

Lastly, we also do not examine our data in the framework of East/West or individualistic/collectivist traits, often used in cross-cultural psychology. Of note, no consistent pattern of cognitive performance appeared between East (Asia) and West (Anglosphere or Europe) regions (Supplementary Figure S1).

## Conclusion

In sum, lasting cultural biases exist on neuropsychological tests in first-generation immigrants with PD despite significant exposure to, and participation in, Canadian culture. While previously identified contributing factors such as education, English proficiency, and verbal nature of the tasks do not account for this bias, we provide compelling evidence that the socio-development and historical context in which individuals are born is a strong and persistent contributor. At a coarse level, being born in Anglosphere countries, which share cultural and historical roots, and similar high levels of economic and social development offers an advantage on cognitive tests that are typically conceived in these regions. A finer and scalable metric of human socio-development borrowed from the field of Development Economics, namely the HIHD, robustly mediates this relationship. Hence, the integration of such societal indices has strong potential to benefit research in cross-cultural psychology. For the current practice of clinical neuropsychology, our findings underscore the need to suspect the presence of cultural bias when assessing immigrants, particularly those originating from countries with low human development index, irrespective of their English proficiency, educational and professional attainment, or length of time since immigration.

## Data Availability

The datasets for this study will not be made publicly available because this consists of clinical data and we did not request permission from the REB to share data outside our institution.

## Ethics Statement

This study conforms to the standards set by the latest revision of the Declaration of Helsinki and was approved by the Research Ethics Board of the University Health Network.

## Author Contributions

MS and MC designed the study, extracted data from clinical charts, interpreted data, and wrote the manuscript. MC performed statistical analyses.

## Conflict of Interest Statement

The authors declare that the research was conducted in the absence of any commercial or financial relationships that could be construed as a potential conflict of interest.

## Footnotes

^1^www.processmacro.org
