# Supplementary material for: Origins Matter: Culture Impacts Cognitive Testing in Parkinson’s Disease
Source: Front Hum Neurosci. 2019 Aug 8;13:269. doi: 10.3389/fnhum.2019.00269 (PMC6694800; doi:10.3389/fnhum.2019.00269)
Supplement: Supplementary file 1 [file Data_Sheet_1.PDF]

Table S1. Number of PD patients per country or birth and median HIHD

Regions/Countries	N	HIHD (median)
Anglosphere	248	
Canada	220	0.502
UK/Ireland	22	0.460
USA	6	0.532
Africa	12	
Egypt	4	0.125
Nigeria	1	0.094
South Africa	4	0.212
Tanzania (includes Zanzibar)	1	0.105
Uganda	2	0.119
Americas/Caribbean	24	
Brazil	1	0.208
Chile	2	0.301
Colombia	3	0.244
Ecuador	2	0.193
El Salvador	2	0.161
Guyana	6	0.284
Jamaica	2	0.255
Peru	2	0.219
Trinidad	3	0.344
Venezuela	1	0.231
Central, South & West Asia	59	
Afghanistan	2	0.080
Bangladesh	2	0.100
India	24	0.117
Iran	9	0.148
Iraq	2	0.137
Lebanon	1	0.247
Pakistan	11	0.111
Sri Lanka	7	0.286
Syria	1	0.190
East & Southeast Asia	32	
Burma (currently Myanmar)	2	0.097
China	9	0.150
Hong Kong	10	0.271
Philippines	5	0.289
Singapore	1	0.303
Vietnam	5	0.154

Table S1 (cont'd). Number of PD patients per country or birth and median HIHD

Europe	40	
Albania	1	0.301
Czechoslovakia (currently Czech Republic and Slovakia)	1	0.387
Denmark	1	0.443
France	1	0.407
Germany	5	0.428
Greece	2	0.260
Italy	8	0.346
Netherlands	1	0.476
Norway	1	0.502
Poland	3	0.338
Portugal	6	0.287
Romania	1	0.260
Serbia (includes Macedonia and Yugoslavia)	3	0.264
Spain	1	0.244
USSR (includes Belarus, Kazakhstan, Lithuania, Russia, and Ukraine)	5	0.384

Supplementary Analyses – Effects of demographic and motor scores on performance biased tasks between groups

Although most demographic and disease-related variables were similar between groups, two variables differed (i.e., older age in the Anglosphere group and higher UPDRS part 3 OFF scores in the International group). Here, we provide supplementary analyses investigating whether age and UPDRS part 3 OFF, as well as other variables known to contribute to performance on cognitive testing irrespective of culture (sex, education) have differential relationships with cognition across groups. For each task identified as culturally biased, we conducted Spearman correlations between performance and continuous variables (age, education, UPDRS part 3 OFF) for each group (Table S2), and a two-way ANOVA (group x sex; Table S3) to test for an interaction. Results do not suggest the presence of differential effects of these variables on performance on biased tests between groups.

Table S2. Spearman correlations between performance on biased tests and age, education and UPDRS part 3 OFF as a function of group (Anglosphere vs. International)

	Education		Age		UPDRS part 3 OFF	
	Anglosphere	International	Anglosphere	International	Anglosphere	International
JLO	.26	.25	-.13	-.15	-.23	-.13
Silhouettes	-.01	.04	-.19	-.16	-.04	-.02
Object Decision	-.09	.03	-.08	-.08	-.04	-.12
Matrix Reasoning	.34	.50[b]	-.18	-.30	-.23	-.16
Category Fluency	.24	.20	-.22	-.23	-.17	-.13
WCST errors	-.30	-.35	.24	.21	.13	.10

Note. a: JLO = Benton Judgement of Line Orientation, WCST = Wisconsin Card Sorting Test

b: The greatest difference is seen on the Matrix Reasoning subtest and education correlations (.16 difference), but the magnitude of the correlations does not differ significantly between groups (z = 1.88 p = .06).

Table S3. Two-way ANOVA between performance on biased tests and group x sex

	Main effect Group	Main Effect Sex	Interaction (group x sex)
JLO	$F(1, 410) = 30.68$, $p<.001$	$F(1, 410) = 18.91$, $p<.001$[b]	$F(1, 410) = 2.98$, $p = .09$
Silhouettes	$F(1, 378) = 50.87$, $p<.001$	$F(1, 378) = 4.91$, $p = .03$	$F(1, 378) = 0.07$, $p = .79$
Object Decision	$F(1, 411) = 74.16$, $p<.001$	$F(1, 411) = 2.72$, $p = .10$	$F(1, 411) = 1.65$, $p = .20$
Matrix Reasoning	$F(1, 408) = 25.94$, $p<.001$	$F(1, 408) = 0.08$, $p = .78$	$F(1, 408) = 0.73$, $p = .39$
Category Fluency	$F(1, 409) = 61.54$, $p<.001$	$F(1, 409) = 10.92$, $p = .001$[b]	$F(1, 409) = 0.07$, $p = .77$
WCSTerrors	$F(1, 407) = 7.21$, $p = .008$	$F(1, 407) = 3.80$, $p = .05$	$F(1, 407) = 3.47$, $p = .06$

Note. a: JLO = Benton Judgement of Line Orientation, WCST = Wisconsin Card Sorting Test

b. Men obtained higher scores on JLO than Women, but lower scores on Category Fluency
