# Supplementary figures and images for: Origins Matter: Culture Impacts Cognitive Testing in Parkinson’s Disease
Source: Front Hum Neurosci. 2019 Aug 8;13:269. doi: 10.3389/fnhum.2019.00269 (PMC6694800; doi:10.3389/fnhum.2019.00269)

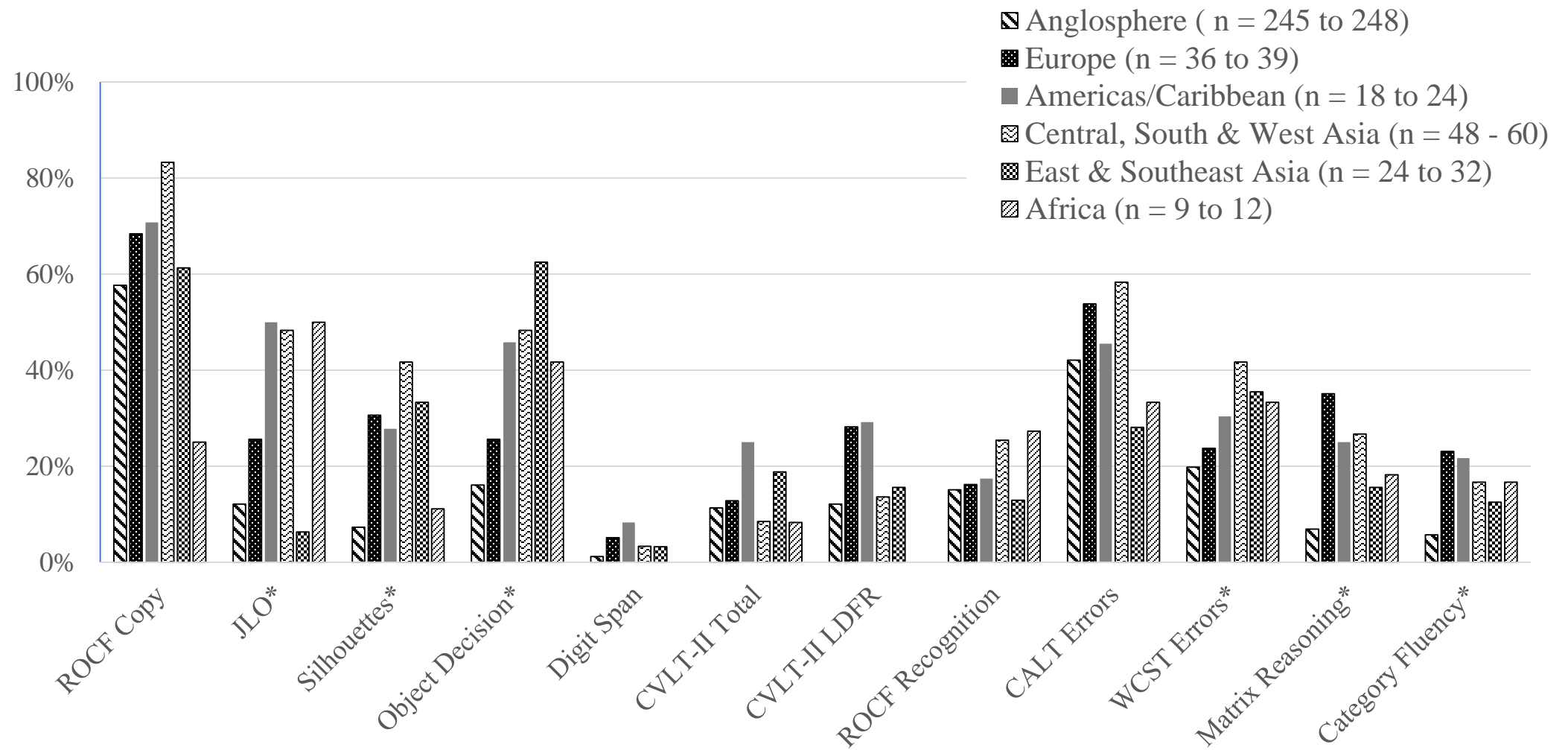

Fig. S1. Frequency of impairment in PD per cognitive tests and world region

Supplement: Supplementary file 2 [file Data_Sheet_2.PDF]
